# Advancements in theranostic applications: exploring the role of fibroblast activation protein inhibition tracers in enhancing thyroid health assessment

**DOI:** 10.1186/s13550-023-01060-8

**Published:** 2023-12-22

**Authors:** Yuhua Wang, Ye Liu, Huixia Geng, Wanchun Zhang

**Affiliations:** 1 Department of Nuclear Medicine, Shanxi Bethune Hospital, Shanxi Academy of Medical Sciences, Tongji Shanxi Hospital, Third Hospital of Shanxi Medical University, Long Cheng Street 99, Xiao Dian District, Taiyuan, 030032 Shanxi China; 2 Tongji Hospital, Tongji Medical College, Huazhong University of Science and Technology, Wuhan, 430030 China

**Keywords:** Fibroblast activation protein, Fibroblast activation protein inhibitors, Thyroid cancer, Positron emission tomography

## Abstract

**Background:**

The diagnostic accuracy of [^18^F]-fluorodeoxyglucose ([^18^F]-FDG) positron emission tomography imaging in accurately identifying thyroid lesions is limited, primarily due to the physiological uptake of normal head and neck tissues and inflammatory uptake in lymph nodes. Since fibroblast activating protein is highly expressed in tumors and largely unexpressed in normal tissues, quinoline-based fibroblast activating protein inhibitors (FAPI) have emerged as promising tools in the diagnosis of cancer and other medical conditions. Several studies have reported on the feasibility and value of FAPI in thyroid cancer.

**Main body:**

In this narrative review, we summarize the current literature on state-of-the-art FAPI positron emission tomography imaging for thyroid cancer and fibroblast activating protein-targeted radionuclide therapy. We provide an overview of FAPI uptake in normal thyroid tissue, thyroid cancer and its metastases. Additionally, we highlight the difference between FAPI uptake and [^18^F]-FDG uptake in thyroid lesions. Furthermore, we discuss the therapeutic value of FAPI in iodine-refractory thyroid cancer.

**Conclusion:**

The utilization of fibroblast activating protein inhibitors in thyroid cancer holds significant promise, offering clinicians valuable insights for more precise diagnose choices and treatments strategies in the future.

## Background

Cancer development occurs within complex environments, encompassing both tumor cells and the surrounding stroma. The "seed and soil" theory, proposed in 1889, laid the foundation for understanding tumor metastasis evolving into the key theoretical framework known as the tumor microenvironment (TME) [1]. The significance of the supporting stroma becomes apparent when the tumor mass exceeds 1–2 mm, constituting a substantial portion of the lesion, often surpassinging the tumor’s own volume [2]. Central to the TME, cancer-associated fibroblasts (CAFs) play a key role in facilitating cancer cell proliferation, augmenting both proliferative and migratory capabilities, and metastatic dissemination. Notably, fibroblast activating protein (FAP), a specific marker for CAFs, exhibits elevated expression within fibroblasts across numerous solid malignant tumors [3, 4]. Within tumor tissues, FAP engages signaling pathways that govern tumor cell invasion and metastasis, stimulating the proliferation and malignant transformation of neighboring epithelial cells, inducing tumor immune escape, participating in neovascularization, etc. Overexpression of FAP in solid malignant tumors correlates with poor overall survival and lymph node metastasis [5]. Leveraging the distinct expression patterns of FAP between normal tissue and tumors, FAP inhibitors (FAPI) with high affinity for FAP have emerged as radioligands for imaging and therapy [6]. The biodistribution of FAPI PET/CT imaging within cancer closely mirrors FAP expression within tissues [4]. Clinically reliable diagnosis of primary tumors, metastatic lesions, and involved lymph nodes is critical for formulating effective treatment strategies, encompassing tumor staging and therapeutic choices. Notably, FAP expression strongly correlates with tumor size, lymph node metastasis, and TNM classification [7–9]. Currently, FAPI shows utility in the diagnosis of diverse cancers including lung, pancreatic, colon, and liver. Moreover, it is being employed in salvage therapy for advanced diseases due to its high contrast attributes. In the context of thyroid papillary carcinoma, which exhibits fibrosis [10], mesenchymal stem cells highly express FAP [11]. This expression correlates with TNM stage and lymph node metastasis [7]. Thus, radionuclide-labeled FAPI also finds applications in differentiated thyroid cancer, especially in cases of iodine-refractory thyroid cancer. This review summarizes the application of cutting-edge FAPI-PET imaging techniques for diagnosing thyroid cancer and its potential in FAP-targeted radionuclide therapy.

## Main text

### FAPI uptake in the normal thyroid

Immunohistochemical studies have demonstrated positive expression of FAP in papillary thyroid carcinoma (PTC), while remaining absent in the follicular epithelium and stromal cells of the normal thyroid [7–9]. According to these findings, the thyroid should not be visibly detected on FAPI imaging, however, a level of controversy persists within the literature regarding this assertion. Notably, the physiological biodistribution of [^177^Lu]-FAPI signifies that FAPI is not involved in thyroid glands [12]. In a study detailing [^68^Ga]-FAPI uptake in healthy tissues of cancer patients, the thyroid gland exhibited low uptake of [^68^Ga]-FAPI, characterized by a median standardized uptake value (SUVmax) of 2.02[13]. This is consistent with the findings of another study [14] where the median SUVmax for [^68^Ga]-FAPI uptake within the thyroid gland of patients with diverse solid tumors was 1.36 ± 0.26. Conversely, an investigation of the physiological tracer uptake of [^18^F]-FAPI revealed evident uptake within the thyroid [6]. The average SUVmax for the thyroid in this study reached 5.7 ± 3.6, surpassing prior observations. It is noteworthy that among the study participants, only seven out of twenty underwent ultrasound and thyroid function examinations. Further examination of [^99m^Tc]-FAPI SPECT/CT imaging showed mild uptake within the thyroid gland, which was comparatively lower than in other organs. Importantly, this uptake demonstrated a decreasing trend as the drug injection time was extended [15]. Dynamic PET/CT scans involving [^68^Ga]-FAPI have also shown a higher SUVmean for the thyroid in comparison to most other organs, yet this value gradually declined over time [16]. Additionally, a gender-based difference was noted in [^68^Ga]-FAPI uptake, with males exhibiting higher uptake during the initial time points when compared to females [16]. Existing studies have suggested distinct biological distribution of [^18^F]-FAPI-42 and [^68^Ga]-FAPI-04 within normal organs. This variation could potentially be related to the different lipophilicities of the chelating agents used in these compounds [17]. In summary, the inconsistencies observed in FAPI uptake across thyroid tissues in the aforementioned studies may stem from multiple factors, including differences in tracers, gender-specific variations and disparities in imaging time.

### FAPI uptake in thyroid cancer and metastatic lesions

Fibrosis is a characteristic feature of papillary thyroid carcinoma. Notably, papillary mesenchymal stem cells within this context exhibit elevated FAP expression compared to their non-carcinogenic thyroid mesenchymal stem cell counterparts [11]. Several studies have suggested the presence of FAPI uptake within thyroid cancer, with a median SUVmax of 3.3[18]. This aligns with the average uptake of [^68^Ga]-FAPI in DTC in another study (SUVmax < 6) [19]. Importantly, the expression of FAP demonstrates good predictive value for external thyroid invasion, BRAF mutation, and lymph node metastasis [20]. In light of these findings, a pertinent question arises: can FAPI imaging serve as a predictive tool for disease prognosis and assist in guiding the management of thyroid cancer?

Following surgery, radioactive iodine therapy, and thyroid hormone suppression, the majority of differentiated thyroid cancer (DTC) patients experience successful outcomes, with over 95% achieving a 20-year survival rate [21]. However, approximately 20% of cases encounter local recurrence, while 10% face distant metastasis [22]. Among these scenarios, around two-thirds of the cases transition into radioiodine-refractory thyroid cancer (RR-DTC) during subsequent follow-up, characterized by a loss of iodine uptake capacity, rapid disease progression, and a 10-year survival rate of less than 10% [22, 23]. FAP expression is also observable within the majority of metastatic and RR-DTC lesions [24, 25]. The extent of FAP expression in the surrounding matrix of these metastatic, recurrent thyroid cancer, and RR-DTC cases correlates with the degree of FAPI uptake. Lesions exhibiting a high level of FAPI uptake show an accelerated short-term growth rate [25].

Several studies have applied FAPI imaging to the exploration of thyroid metastases and have demonstrated a high detection rate [25, 26]. When focusing on recurrence and metastases, local recurrence exhibits the highest degree of FAPI uptake, surpassing that of lymph nodes, bones, pleura, and the lungs [27]. Furthermore, the SUVmax within positive lesions shows a positive correlation with lesion size [27]. FAPI PET/CT imaging offers distinct advantages over FDG in the context of TNM staging for nasopharyngeal carcinoma [28, 29]. Therefore, FAPI PET/CT imaging might hold potential in identifying the TNM stage of thyroid cancer.

### Comparing FAPI and [^18^F]-FDG uptake

The early detection of cervical lymph node metastases is crucial in the management of DTC. When the tumor is situated in the upper third of the thyroid lobe or if the count of central lymph node metastases exceeds three, vigilance must extend to the lateral compartment due to its susceptibility to lateral lymph node metastasis [30]. Notably, [^18^F]-fluorodeoxyglucose ([^18^F]-FDG) PET/CT imaging often encounters challenges in accurately identifying lesions; this is primarily attributed to the physiological uptake of normal head and neck tissues and the inflammatory uptake of lymph nodes [17]. Considering that FAPI is a novel PET tracer in cancer imaging, numerous studies have undertaken comparisons between FAPI and FDG, with several directly comparing the two radioactive tracers in the contest of thyroid cancer. In delineating neck lesions, the sensitivity of [^68^Ga]-FAPI PET/CT was 83%, surpassing the 65% achieved by [^18^F]-FDG [24]. The positive predictive value (PPV) of [^68^Ga]-FAPI for diagnosing lymph node metastases was 86.49, which outperformed the 68.09 recorded for [^18^F]-FDG [31]. A comparison between [^68^Ga]-FAPI and [^18^F]-FDG imaging in thyroid postoperative patients revealed that FAPI exhibited a higher count of displayed lesions compared to FDG. Additionally, the newly identified lesions were primarily cervical lymph nodes, boasting a sensitivity of 83% [24]. Another study [32] even identified inguinal lymph node metastasis using FAPI imaging, which eluded detection by [^18^F]-FDG imaging. Furthermore, the SUVmax of [^68^Ga]-FAPI in DTC metastatic lateral cervical lymph nodes of DTC surpassed that of [^18^F]-FDG [24]. This trend was echoed in the context of lymphatic lesions, where the SUVmax of [^18^F]-FAPI exceeded that of [^18^F]-FDG [27]. However, a separate study reported no significant difference in SUVmax values for lymph node metastases between [^68^Ga]-FAPI and [^18^F]-FDG [31]. Despite variations in SUVmax values reported by these studies, the diagnostic efficiency of FAPI imaging remains robust due to its favorable target-background profile.

Research indicates that [^68^Ga]-FAPI imaging is not inferior to [^18^F]-FDG when applied to patients with recurrent papillary thyroid carcinoma who were previously treated with radioiodine [33]. For patients experiencing hematobiochemical recurrence in DTC, the SUVmax values of recurrent lesions detected by [^68^Ga]-FAPI outperform those of [^18^F]-FDG, and this difference is statistically significant [33]. A similar trend was also noted in cases of local recurrence lesions in DTC with associated biochemical elevation, as assessed through both [^18^F]-FAPI and [^18^F]-FDG [27]. Therefore, FAPI imaging can prove useful for DTC patients with elevated Tg or TgAb levels.

In cases of distant metastases, lungs and bones are common sites of metastasis in DTC patients. The PPV of [^68^Ga]-FAPI in diagnosing bone metastases was found to be significantly higher compared to [^18^F]-FDG [31]. This study revealed that the difference in SUVmax values for bone metastases was markedly higher when measured with [^68^Ga]-FAPI than with [^18^F]-FDG. However, in terms of lung metastases, this difference was not significant [31]. Nevertheless, another study [24] reported a contrasting conclusion, asserting that the [^68^Ga]-FAPI-derived SUVmax was significantly higher in lung metastases than one derived from [^18^F]-FDG, but with no significant difference observed in bone metastases. Adding to the complexity, another study [27] showed that the median SUVmax and median TBR of lung lesions were lower when measured with [^18^F]-FAPI in comparison to [^18^F]-FDG. Given the divergent findings reported in these studies, it is evident that further research should be conducted to attain a clearer understanding of these variations and their underlying mechanisms.

Brain metastasis stemming from DTC is associated with a poor prognosis, with an incidence of approximately 1.09% and an increasing trend in recent years [34]. The application of [^18^F]-FDG in brain metastasis cases is hindered by the elevated brain background activity. Due to its higher image contrast, [^68^Ga]-FAPI has the capability to identify brain lesions that may go unnoticed in [^18^F]-FDG imaging [29, 32]. The heightened sensitivity of FAPI imaging in comparison to [^18^F]-FDG presents a promising attribute for evaluating brain metastases.

Research findings highlight that FAPI imaging has the potential to identify more thyroid cancer-associated metastases than [^18^F]-FDG [32]. Moreover, it can be utilized in conjunction with [^18^F]-FDG, leading to the enhanced detection of metastatic foci [33]. A noteworthy case study involving a DTC patient following radioiodine therapy demonstrated negative uptake in liver and bone metastases through [^18^F]-FDG imaging, while [^68^Ga]-FAPI imaging showed mild uptake in the same regions [35]. This observation suggests that [^68^Ga]-FAPI may offer valuable insights for the restaging of metastatic DTC cases. Of note, FAPI imaging primarily illuminates the tumor stroma, while FDG imaging visualizes tumor cell glycolysis [2]. Therefore, FAPI imaging presents substantial advantages over FDG imaging in detecting thyroid cancer metastases.

### FAPI in other thyroid diseases

It is important to note that FAP expression can be increased not only within malignant lesions but also in non-cancerous pathologies. Focal thyroid uptake of FAPI can also signify benign conditions. For instance, the presence of thyroiditis, can lead to FAP expression in fibroblasts surrounding clusters of lymphocyte infiltration [36]. Furthermore, diffuse thyroid uptake of FAPI is commonly associated with thyroiditis. Liu et al. [14] reported diffuse [^68^Ga]-FAPI uptake in the thyroid among 39 out of 815 (4.8%) patients, and this uptake was nearly always associated with thyroiditis. The median SUVmax was 4.15[14]. Another study reported similar levels of [^68^Ga]-FAPI SUVmax in thyroiditis cases [37]. Immune-related thyroiditis (irT) emerges in tumor patients after treatment with immune checkpoint inhibitors (ICIs). This condition is characterized by early onset thyrotoxicosis followed by a rapid progression to hypothyroidism. This becomes especially rapid in cases where a combination of anti-CTLA-4 and anti-PD1 therapy is employed [38]. When these patients undergo [^68^Ga]-FAPI PET/CT imaging, the thyroid gland exhibits diffuse uptake [14], with the highest SUVmax reported in the literature reaching 23.5 [39]. Importantly, the thyroid uptake tends to be relatively low when the PET/CT scan interval is shorter after immunosuppressive treatment [14].

In addition to thyroiditis, it is worth highlighting that thyroid lymphoma can also show diffuse abnormal uptake of [^68^Ga]-FAPI [40]. Moreover, benign thyroid lesions exhibiting increased [^68^Ga]-FAPI uptake are common. For instance, in one study, the median SUVmax for such lesions was 3.64 [18]. In a case of thyroid nodular goiter characterized by fibrosis and calcification, [^68^Ga]-FAPI uptake was observed with a SUVmax of 7.7 and a TBR of 5.5[41]. Even follicular thyroid adenoma can exhibit elevated [^68^Ga]-FAPI uptake [42], potentially attributed to fibrous tissue hyperplasia within the tumor. Consequently, it is imperative to exercise caution and consider these factors when interpreting FAPI uptake in the thyroid.

### Correlation of FAPI with genetic and serological indicators

Numerous studies have explored the correlation between [^68^Ga]-FAPI imaging and serological markers. The degree of [^68^Ga]-FAPI uptake appears to lack a connection with the severity of thyroiditis, levels of TSH, and TPOAb [14]. Fu et al. [43] observed that FAPI uptake did not significantly vary among patients with various Tg levels. This led them to speculate that FAP expression might not be closely associated with Tg levels. Likewise, another study showed that serum TSH, Tg and TgAb levels in patients did not markedly impact the uptake of [^18^F]-FAPI in thyroid cancer lesions [27]. However, the relationship between FAPI imaging and Tg levels remains a point of contention. Chen et al. [25] reported that patients with intense FAPI uptake lesions exhibited elevated Tg levels, while those with lower uptake of [^68^Ga]-FAPI displayed lower Tg levels. The positivity rate of [^68^Ga]-FAPI seemed to rise in tandem with increasing Tg levels, reaching 100% when Tg exceeded 300 [33]. Moreover, the TBR of [^18^F]-FAPI appeared to vary across different biomarker levels in one study [27]. Hence, the interplay between FAP expression and Tg or TgAb levels requires further exploration.

In addition to serological markers, gene expression appears to influence FAPI uptake. It was reported [44] that the prevalence of the BRAFV600E mutation is significantly associated with elevated FAP expression in human PTCs. Specifically, PTCs carrying the BRAF mutation showed higher FAP expression both within tumor cells and in the surrounding stromal cells. Consequently, FAPI uptake was notably higher in the BRAFV600E mutation group when compared to the wild-type group [27].

In conclusion, it is evident that both blood biochemistry and gene expression can potentially influence FAPI imaging outcomes.

### Therapeutic effect of FAPI on thyroid cancer

Radioiodine treatment is the mainstay adjuvant approach in the management of intermediate and high-risk differentiated thyroid cancer. However, as thyroid cancer advances, a decrease in sodium iodine transporters and an upregulation of glucose transporter-1 (GLUT1) often lead to the development of RR-DTC [45]. In response to this challenge, multikinase inhibitors (MKIs) have emerged as therapeutic options for such patients. Nevertheless, a subset of individuals might not experience effective responses to MKIs, and a considerable proportion of them encounter treatment-related adverse events (AEs).

Thanks to its high-contrast, FAP is recognized as an appealing target for radionuclide therapy [46, 47]. The use of FAPI-based radio-ligands to target the TME has been explored in certain patients with advanced cancer, displaying promising feasibility [48]. FAP expression has been observed in RR-DTC lesions [24, 25], and FAPI imaging has shown concentrated radioactive uptake within these lesions [25]. Based on these observations, [^177^Lu]-FAPI has recently been utilized in treating RR-DTC [12, 49]. [^177^Lu]-FAPI whole-body scintigraphy has revealed robust radiotracer uptake within metastatic DTC lesions post-therapy. Notably, a patient achieved stable disease, coupled with a reduction in analgesic scores [49]. A significant reduction in Tg levels among 15 RR-DTC patients was also observed following treatment, and none experienced severe grade III/IV hematological, renal, or hepatotoxicity adverse events [12]. These findings suggest that [^177^Lu]-FAPI therapy holds potential as a valid treatment option for advanced DTC patients.

## Conclusions

The role of [^18^F]-FDG imaging in the diagnosis and preoperative staging of thyroid cancer has been limited. Current literature suggests that FAPI labeled with [^68^Ga] or [^18^F] offers superior diagnostic value for thyroid cancer and its metastases compared to [^18^F]-FDG. As a result, there is potential for the application of FAPI imaging in the preoperative staging of thyroid cancer. Moreover, the substantial FAP expression in thyroid metastatic lesions suggests that FAP is a potential therapeutic target. In this context, the use of [^177^Lu]-FAPI holds promise to emerge as a significant treatment avenue for patients with RR-DTC.

## Data Availability

Not applicable.

## Abbreviations

[^18^F]F-FDG: [^18^F]-fluorodeoxyglucose
[^68^Ga]-FAPI: Gallium 68-labeled fibroblast activation protein inhibitor
PET/CT: Positron emission tomography/computed tomography
TME: Tumor microenvironment
DTC: Differentiated thyroid carcinoma
PTC: Papillary thyroid carcinoma
SUV: Standardized uptake value
RR-DTC: Iodine-refractory thyroid cancer
MKIs: Multikinase inhibitors
AEs: Adverse events
CAFs: Cancer-associated fibroblasts
TBR: Target-to-blood ratio
PPV: Positive predictive value
TSH: Thyroid stimulating hormone
Tg: Thyroglobulin
TgAb: Thyroglobulin antibody
TPOAb: Thyroid peroxidase antibody
GLU-1: Glucose transporter-1
CTLA4: Cytotoxic T-lymphocyte antigen 4
PD1: Programmed cell death protein 1
